# The impact of growth mindset on subjective wellbeing in elementary school students: a moderated mediation model

**DOI:** 10.3389/fpsyg.2025.1595422

**Published:** 2025-07-09

**Authors:** Lingyi Peng, Huohong Chen, Wei Liang, Zifu Shi, Qiaoping Li

**Affiliations:** ^1^Cognition and Human Behavior Key Laboratory, Hunan Normal University, Changsha, Hunan, China; ^2^Hunan First Normal University, Changsha, Hunan, China; ^3^Hunan Mass Media Vocational and Technical College, Changsha, Hunan, China

**Keywords:** growth mindset, subjective wellbeing, academic self-efficacy, perceived teacher support, elementary school students

## Abstract

**Introduction:**

This study examines the relationship between growth mindset and subjective wellbeing among elementary school students, with a focus on the mediating role of academic self-efficacy and the moderating role of perceived teacher support.

**Methods:**

Utilizing the cluster sampling method, a sample of 1,740 elementary school students completed measures assessing growth mindset, subjective wellbeing, academic self-efficacy, and perceived teacher support.

**Results:**

The results show that: (1) growth mindset positively predicts elementary school students' subjective wellbeing; (2) academic self-efficacy partially mediates the association between growth mindset and elementary school students' subjective wellbeing; and (3) perceived teacher support moderates the first stage of the mediation pathway, such that the positive effect of growth mindset on elementary school students' academic self-efficacy is stronger under high levels of perceived teacher support.

**Discussion:**

These findings indicate that growth mindset influences elementary school students' subjective well-being both directly and indirectly through academic self-efficacy. Moreover, the impact of growth mindset on academic self-efficacy is stronger among elementary school students with higher perceived teacher support.

## 1 Introduction

All human activities serve not only to sustain existence but, more importantly, to attain wellbeing (Diener and Ryan, 2009). A positive life evaluation consititutes an integral component of a flourishing life (Zhang and Zheng, 2004). Subjective wellbeing is defined as an individual's cognitive appraisal and emotional experience of overall life satisfaction (Diener, 1984). It comprises three key dimensions: positive affect, negative affect, and life satisfaction (Diener et al., 2015). Research has indicated that childhood wellbeing can alleviate stress during individual development and decrease the risks to physical and mental health (Wang et al., 2019). However, recent surveys reveal an unfavorable status quo regarding elementary school students' subjective wellbeing, with an overall declining trajectory (Fan et al., 2025). Cognitive Behavior Theory (Beck et al., 1979) posits that core beliefs constitute significant predictors of subjective wellbeing. Growth mindset, as a positive cognitive belief, has been empirically linked to enhance subjective wellbeing (Ortiz Alvarado et al., 2019; Zhao et al., 2024). Considering that elementary school is a critical period for the development of a growth mindset, students at this stage begins to form core self-beliefs based on their educational experiences (Dweck, 2002; Muenks et al., 2018). Therefore, this study further examines the impact of growth mindset on elementary school students' subjective wellbeing and its underlying psychological mechanisms, aiming to provide empirical support for enhancing their subjective wellbeing.

### 1.1 Growth mindset and subjective wellbeing

Growth mindset reflects an individual's belief in the malleability of their abilities, as opposed to a fixed mindset (Dweck, 2006). Mindset theory (Dweck, 2006) posits that, individuals with growth mindset tend to perceive setbacks or failures as opportunities for learning and skill development, enabling them to maintain positive emotions comparable to those experienced during success even in the face of failure. In contrast, individuals with a fixed mindset attribute failure to inherent ability deficits, viewing it as a threat to their self-worth. This often leads to feelings of helplessness, avoidance behaviors, and increased susceptibility to negative emotions such as anxiety and depression (Schroder et al., 2015). Within this framework, growth mindset is posited as a key predictor of subjective wellbeing. Empirical studies have further substantiated this perspective. For example, Zhao et al. (2024) demonstrated through research on high school students that those endorsing a growth mindset exhibited higher levels of subjective wellbeing, and this finding was further validated in a college student population (Ortiz Alvarado et al., 2019). Elementary school is widely recognized as a critical window for cultivating a growth mindset (Dweck, 2002; Muenks et al., 2018). Empirical studies have further established that elementary school students' growth mindset directly influences their life satisfaction and affective states (Diao et al., 2020; Huang et al., 2023). Collectively, this study suggests that growth mindset may positively predict elementary school students' subjective wellbeing.

### 1.2 The role of academic self-efficacy

Academic self-efficacy refers to an individual's judgment and confidence in their ability to succeed academically (Wang et al., 2016). Self-Determination Theory (Deci and Ryan, 2000) posits that growth mindset enhances individuals' confidence in achieving academic goals by fulfilling their psychological needs, thereby promoting subjective wellbeing. Consequently, academic self-efficacy may serve as a critical mediator in the relationship between growth mindset and subjective wellbeing.

On the one hand, growth mindset is closely associated with individuals' academic self-efficacy. Research has demonstrated that growth mindset enhances academic self-efficacy among elementary school students (Diao et al., 2020), a finding that has been replicated in experimental studies with middle school students (Zhao et al., 2023). Furthermore, cross-cultural studies have shown that the positive effect of growth mindset on academic self-efficacy exhibits stability across diverse cultural contexts (Chen and Pajares, 2010). On the other hand, academic self-efficacy significantly promotes for individuals' subjective wellbeing. Research has shown that high academic self-efficacy enhances life satisfaction among university students, thereby increasing their subjective wellbeing (Seo et al., 2018), a finding that has also been established among high school students (Cikrikci and Odaci, 2016). Studies focusing on elementary school students further reveal that academic self-efficacy not only positively predicts positive affect but also reduces negative affect, demonstrating a dual promotive effect on subjective wellbeing (Guo et al., 2019). Taken together, these findings suggest that growth mindset may indirectly influence elementary school students' subjective wellbeing through which growth mindset indirectly improves.

### 1.3 The role of perceived teacher support

Perceived teacher support refers to students' perceptions of their teachers' attitudes and behaviors toward their academic lives (Babad, 1990), and is generally regarded as a crucial contextual factor influencing students' cognitive development. The Theory of Growth Mindset-Situation Interaction (Yeager et al., 2019) posits that supportive environments are essential for translating growth mindset into positive cognitions and behaviors when individuals actively confront challenges or setbacks. According to this theory, the relationship between growth mindset and academic self-efficacy may vary depending on perceived teacher support.

During elementary school, as curriculum difficulty and academic workload increase simultaneously, students face growing academic challenges and pressure (Zhang et al., 2019). Among them, students with a growth mindset tend to perceive academic challenges as opportunities for growth and persistently invest effort (Dweck, 2006). This positive coping strategy helps enhance their confidence in overcoming difficulties and achieving goals, thereby enhancing their academic self-efficacy. In contrast, students with a fixed mindset may attribute academic setbacks to inherent ability deficits, exhibiting feelings of incompetence and maladaptive failure responses (Dweck and Yeager, 2019), which undermines their academic self-efficacy. The positive effects of a growth mindset on learning cognition and outcomes depend on the strength of environmental support (Yeager et al., 2019). When perceived teacher support is high, students with growth mindset can effectively reframe setbacks through sufficient cognitive strategy guidance and developmental feedback provided by teachers. This enables them to translate the belief that “effort fosters ability development” into tangible academic success experiences, thereby enhancing their academic self-efficacy (Cai et al., 2025; Yeager et al., 2019; Zhang et al., 2019). Conversely, when perceived teacher support is low, students with growth mindset struggle to validate the effectiveness of their efforts due to insufficient guidance and encouragement. As a result, their belief in the malleability of ability fails to translate into successful experiences. These disconnect between belief and experiential validation exacerbates frustration and ultimately undermines their academic self-efficacy (Wang and Degol, 2016; Yeager et al., 2019; Yu et al., 2022). Collectively, these findings suggest that perceived teacher support may function as a moderating factor in the relationship between growth mindset and academic self-efficacy.

### 1.4 The current study

**Figure 1:**
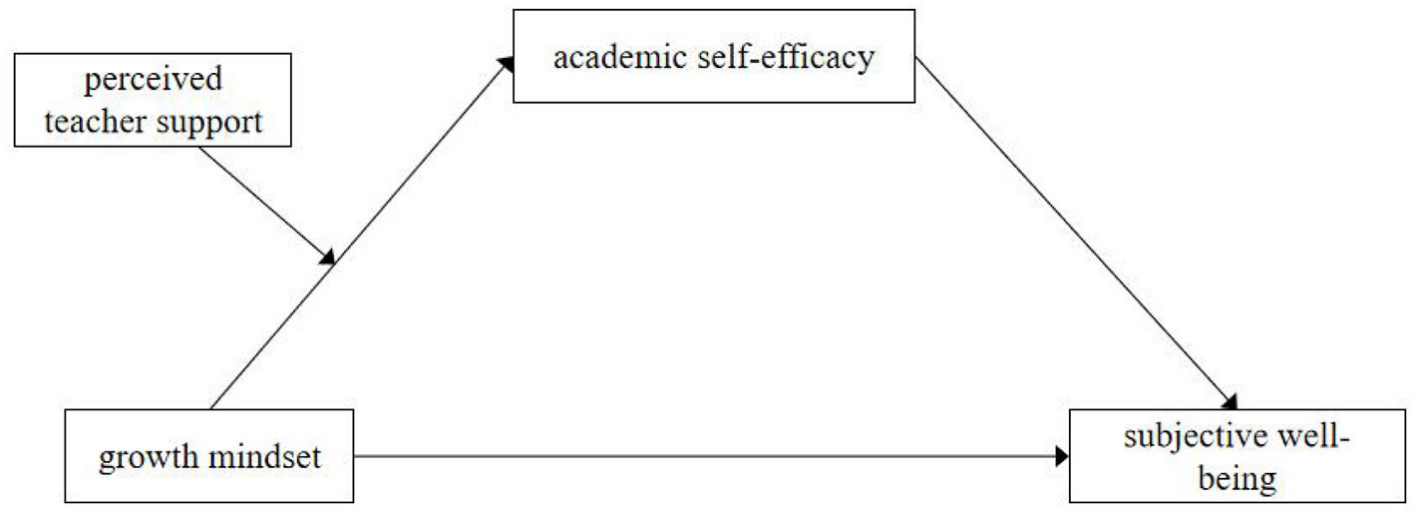
Hypothesized conceptual model.

Building on prior research and theoretical frameworks, this study proposes a moderated mediation model (see Figure 1) to examine the impact of growth mindset on subjective wellbeing among elementary school students, with academic self-efficacy as a mediating factor and perceived teacher support as a moderating variable. The following hypotheses are proposed:

H1: Growth mindset positively predicts elementary school students' subjective wellbeing.

H2: Academic self-efficacy mediates the relationship between growth mindset and elementary school students' subjective wellbeing.

H3: Perceived teacher support moderates the effect of growth mindset on elementary school students' academic self-efficacy.

## 2 Method

### 2.1 Participants

Participants were recruited from grades 4 to 6 across seven elementary schools in H Province. The study was approved by the ethics committee of the authors' university. Using a cluster sampling method, data were collected through group administration in classroom settings, with questionnaires collected immediately after completion. Prior to data collection, informed consent was obtained from both the children and their parents, and confidentiality of responses was assured. Participants were informed that questionnaire completion was voluntary and that they could withdraw from the study at any time. A total of 1,815 questionnaires were distributed. After excluding invalid responses (e.g., random answering patterns or questionnaires with more than 10% missing data), 1,740 valid questionnaires were retained, yielding a response rate of 95.87%. The final sample consisted of 871 boys (50.1%) and 869 girls (49.9%). By grade level, there were 588 fourth graders (33.8%), 530 fifth graders (30.4%), and 622 sixth graders (35.8%). Geographically, 584 students (33.5%) were from urban areas, while 1,156 students (66.5%) were from rural areas.

### 2.2 Measures

#### 2.2.1 Growth mindset scale

The growth mindset sub-scale from the implicit theories of intelligence scale (Dweck, 1999) was used to assess participants' growth mindset. This sub-scale consists of four items. Responses were recorded on a 6-point Likert scale, ranging from 1 (strongly disagree) to 6 (strongly agree). A mean score was calculated across all items, with higher scores indicating a stronger growth mindset. This scale has been widely used in previous studies involving elementary school populations and has demonstrated strong reliability and validity (Cai et al., 2024). In the current study, the growth mindset sub-scale showed a Cronbach's α coefficient of 0.849. The confirmatory factor analysis demonstrated excellent model fit indices: χ^2^*/df* = 1.618, CFI = 1, TLI = 0.997, RMSEA = 0.019, SRMR = 0.004.

#### 2.2.2 Subjective wellbeing scale

The subjective wellbeing scale for children and adolescents, originally developed by Campell et al. (1976) and revised by Dong and Lin (2011), was used to measure subjective wellbeing. This scale consists of nine items across two dimensions: the Affective Balance Index and Life Satisfaction. Responses were recorded on a 7-point Likert scale, ranging from 1 (indicating negative affect or dissatisfaction) to 7 (indicating positive affect or satisfaction). A composite score was calculated by averaging the mean score of the affective balance index (weighted as 1) and the life satisfaction score (weighted as 1.1). Higher scores indicate stronger subjective wellbeing. The scale has been widely used in previous studies involving elementary school students and has demonstrated strong reliability and validity (Ju et al., 2023). In the current study, the scale demonstrated a Cronbach's α coefficient of 0.725. The confirmatory factor analysis indicated a moderately acceptable model fit: χ^2^*/df* = 8.577, CFI = 0.960, TLI = 0.916, RMSEA = 0.066, SRMR = 0.055.

#### 2.2.3 Academic self-efficacy scale

The academic self-efficacy sub-scale from the patterns of adaptive learning scales (Midgley et al., 2000) was used to assess participants' academic self-efficacy. This sub-scale consists of six items, with responses recorded on a 5-point Likert scale ranging from 1 (not at all true) to 5 (completely true). A mean score was calculated across all items, with higher scores indicating greater academic self-efficacy. This sub-scale has been widely used in previous studies involving elementary school populations and has demonstrated strong reliability and validity (Zhen et al., 2015). In the current study, the academic self-efficacy sub-scale showed a Cronbach's α coefficient of 0.748. The confirmatory factor analysis demonstrated excellent model fit indices: χ^2^*/df* = 1.980, CFI = 0.996, TLI = 0.993, RMSEA = 0.024, SRMR = 0.011.

#### 2.2.4 Perceived teacher support scale

The perceived teacher support sub-scale from the school climate scale for adolescents (Jia et al., 2009) was used to measure participants' perceptions of teacher support. The scale consists of seven items, and responses were recorded on a 4-point Likert scale, ranging from 1 (never) to 4 (always). A mean score was calculated across all items, with higher scores indicating greater perceived teacher support. This sub-scale has been widely used in previous studies involving elementary school populations and has demonstrated strong reliability and validity (Hou et al., 2018). In the current study, the perceived teacher support sub-scale showed a Cronbach's α coefficient of 0.81. The confirmatory factor analysis showed acceptable model fit indices: χ^2^*/df* = 6.248, CFI = 0.986, TLI = 0.971, RMSEA = 0.055, SRMR = 0.014.

#### 2.2.5 Data processing and analysis

Confirmatory factor analysis was conducted using Mplus 8.0, followed by descriptive statistics and correlation analyses in SPSS 26.0, and moderated mediation analysis using PROCESS v3.5 (Hayes, 2013).

## 3 Results

### 3.1 Common method bias test

Since all data in this study were collected through self-reports, common method bias was a potential concern. To mitigate this issue, procedural controls were implemented, including the use of validated and reliable measurement tools and emphasizing confidentiality to participants. Statistically, Harman's single-factor test was conducted to assess common method bias. The results revealed six factors with eigenvalues >1, and the first factor accounted for 21.72% of the variance, which is below the critical threshold of 40%. This indicates that common method bias was not a significant issue in this study.

### 3.2 Descriptive statistics and correlation analysis

Independent samples *t*-tests revealed that girls reported significantly higher levels of perceived teacher support than boys (*t* = 2.35, *p* < 0.05). Urban students scored significantly higher than rural students on growth mindset (*t* = 5.54, *p* < 0.001), perceived teacher support (*t* = 8.58, *p* < 0.001), and subjective wellbeing (*t* = 7.06, *p* < 0.001). One-way ANOVA results indicated significant grade-level differences in academic self-efficacy (*F* = 4.10, *p* < 0.05) and subjective wellbeing (*F* = 3.21, *p* < 0.05), but no significant grade-level differences were found for growth mindset (*F* = 1.56, *p* > 0.05) or perceived teacher support (*F* = 0.95, *p* > 0.05).

Table 1 presents the means, standard deviations, and Pearson correlation coefficients for all variables. Pearson correlation analysis revealed that growth mindset was significantly positively correlated with subjective wellbeing (*r* = 0.18, *p* < 0.001) and academic self-efficacy (*r* = 0.29, *p* < 0.001). Subjective wellbeing was also significantly positively correlated with academic self-efficacy (*r* = 0.20, *p* < 0.001). Perceived teacher support showed significant positive correlations with growth mindset, subjective wellbeing, and academic self-efficacy (*r* = 0.17–0.43, *p* < 0.001).

**Table 1:** Descriptive statistics of variables and Pearson correlation matrix (*N* = 1,740).

**Variable**	***M* ±*SD***	**1**	**2**	**3**
1. Growth mindset	4.40 ± 1.23			
2. Subjective wellbeing	5.18 ± 1.34	0.18^***^		
Academic self-efficacy	2.88 ± 0.84	0.29^***^	0.20^***^	
Perceived teacher support	2.43 ± 0.77	0.17^***^	0.26^***^	0.43^***^

*** *p* < 0.001.

### 3.3 Growth mindset and elementary school students' subjective wellbeing: testing a moderated mediation model

A moderated mediation analysis was conducted in two stages (Wen and Ye, 2014). First, a simple mediation model was tested, followed by a moderated mediation model. The non-parametric percentile Bootstrap method was employed, with 5,000 resamples to calculate 95% confidence intervals. Prior to conducting the mediation and moderated mediation analyses, all variables were standardized. Furthermore, based on the preceding analysis results, gender, grade level, and school location were included as control variables in the subsequent analyses (Wen, 2017).

**Step 1:** Examining the mediating role of academic self-efficacy (Model 4). The results showed that growth mindset significantly and positively predicted both subjective wellbeing (*c* = 0.16, *p* < 0.001) and academic self-efficacy (*a* = 0.28, *p* < 0.001). When both growth mindset and academic self-efficacy were included as predictors of subjective wellbeing, growth mindset (*c'* = 0.12, *p* < 0.01) and academic self-efficacy (*b* = 0.15, *p* < 0.001) remained significant positive predictors. The indirect effect (ab) was 0.04, with Boot *SE* = 0.01, and the 95% confidence interval of [0.03, 0.06], which does not include zero. This indicates that academic self-efficacy partially mediates the relationship between growth mindset and subjective wellbeing. The proportion of the indirect effect (*ab*) to the total effect (*c*) was 25% (0.04/0.16).

**Figure 2:**
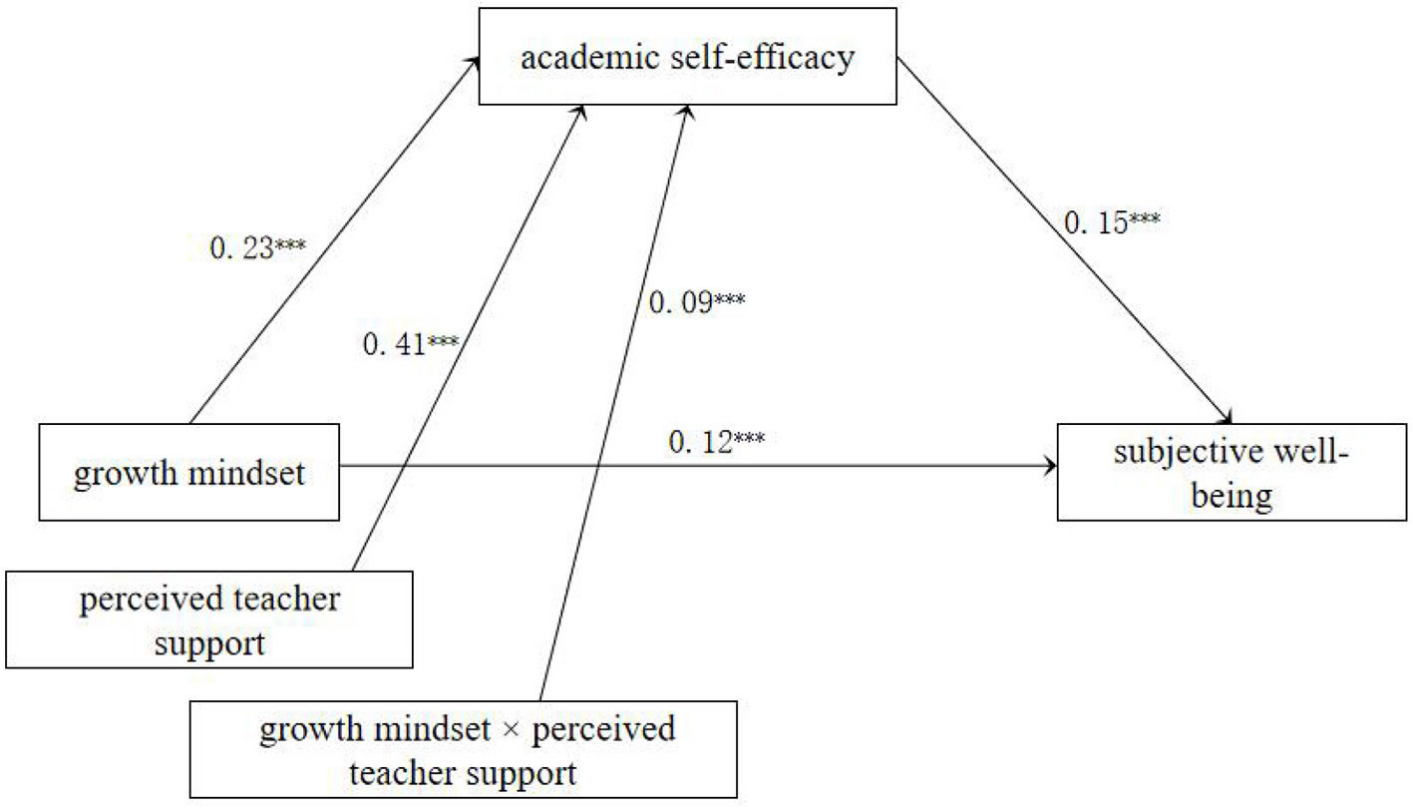
Moderated mediation effect test (*N* = 1,740). ***p < 0.001.

**Step 2:** After controlling for gender, grade level, and school location, perceived teacher support was incorporated into the model to examine the moderated mediation effect using Model 7. The results (see Figure 2) indicated that growth mindset (β = 0.23, *p* < 0.001) and perceived teacher support (β = 0.41, *p* < 0.001) significantly predicted academic self-efficacy. Additionally, the interaction term between growth mindset and perceived teacher support was also significant (β = 0.09, *p* < 0.001). This suggests that perceived teacher support moderates the first half of the mediation pathway (growth mindset → academic self-efficacy → subjective wellbeing).

**Figure 3:**
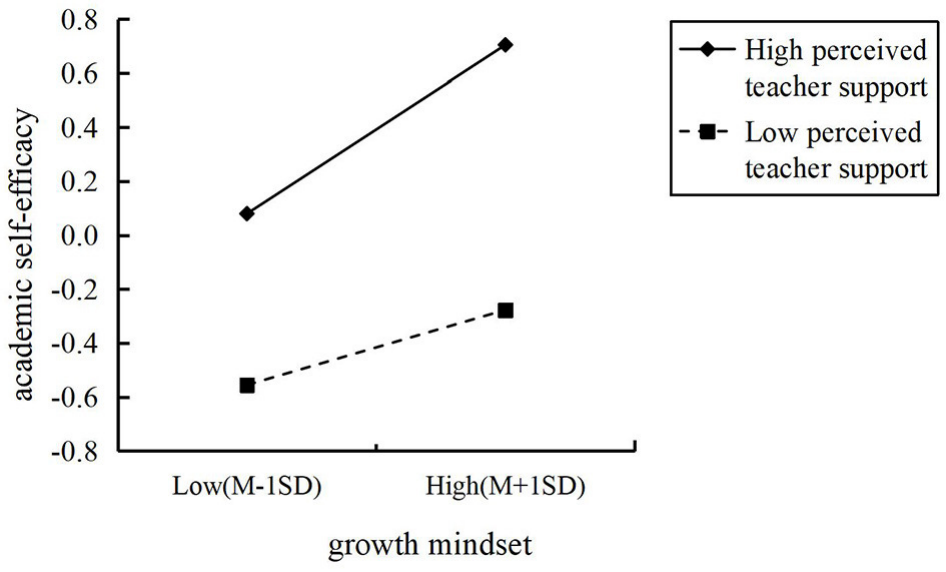
The moderating role of perceived teacher support on the relationship between growth mindset and elementary school students' academic self-efficacy.

To further examine the moderating effect, an interaction plot was created for perceived teacher support (see Figure 3). The slopes of the lines in Figure 3 reflect the magnitude of the effect of growth mindset on academic self-efficacy. Simple slope tests revealed that for students with high perceived teacher support, academic self-efficacy showed a significant upward trend as growth mindset increased (*b*_*simple*_ = 0.31, *t* = 11.03, *p* < 0.001). For students with low perceived teacher support, growth mindset still significantly predicted academic self-efficacy, but the effect was notably weaker (*b*_*simple*_ = 0.14, *t* = 4.89, *p* < 0.001).

## 4 Discussion

This study, through the construction of a moderated mediation model, elucidates the relationship between growth mindset and elementary school students' subjective wellbeing, as well as its underlying mechanisms. First, it clarifies how growth mindset operates—by influencing subjective wellbeing through the mediating role of academic self-efficacy. Second, it examines under what conditions this effect occurs—specifically, the moderating role of perceived teacher support. The findings not only deepen our understanding of the mechanisms underlying subjective wellbeing but also provide important theoretical and practical guidance for enhancing subjective wellbeing among elementary school students.

### 4.1 The impact of growth mindset on elementary school students' subjective wellbeing

The present study found that growth mindset significantly and positively predicted subjective wellbeing among elementary school students, supporting H1. This indicates that, similar to high school and university students, elementary school students with growth mindsets believe in the malleability of their abilities (Dweck, 2006). This belief motivates intrinsic learning drive (Yeoun and Seek, 2018) and facilitates the accumulation of positive emotional experiences, thereby enhancing subjective wellbeing (Blackwell et al., 2007; Yeager et al., 2019). Furthermore, as a critical protective factor for students' mental health (Blackwell et al., 2007), growth mindset plays a significant role in guiding students to adopt adaptive strategies when facing emotional difficulties, adversity, and challenges (Schroder et al., 2015). Students with growth mindset are more likely to respond to academic setbacks and stress with a positive attitude, viewing failures, and challenges as opportunities for growth (Dweck, 2006). By engaging in self-improvement, taking remedial actions (Lam and Zhou, 2020), or employing effective emotion regulation strategies (Schroder et al., 2015), they can enables sustained wellbeing even in the face of failure (Mullarkey and Schleider, 2020). Remarkably, they may even experience increased wellbeing when confronted with challenges (Dweck, 2006).

### 4.2 The mediating role of academic self-efficacy

The current study further revealed that academic self-efficacy served as a significant mediating mechanism in the relationship between growth mindset and elementary school students' subjective wellbeing, confirming H2. This finding also aligns with Self-Determination Theory (Deci and Ryan, 2000), suggesting that growth mindset can enhance students' academic self-efficacy by satisfying their psychological needs, thereby subsequently elevating their subjective wellbeing.

On the one hand, elementary school students possessing a growth mindset view ability development as a dynamic process improvable through effort (Dweck, 2006). Consequently, they demonstrate a greater inclination to proactively select and embrace challenges rather than avoid difficulties or rely excessively on others (Blackwell et al., 2007). This approach not only bolsters students' sense of self-determination and mastery (Burnette et al., 2020; Deci and Ryan, 2000), thereby reducing the risk of learned helplessness, but also directly translates the fulfillment of their autonomy needs into robust intrinsic motivation and sustained academic engagement. This, in turn, strengthens learning confidence and fosters more stable experiences of wellbeing (Guo et al., 2019). On the other hand, a growth mindset encourages elementary school students to confront challenges directly. Through persistent effort and strategic adaptation, they cultivate successful experiences that foster a robust sense of competence and positive self-evaluation. This process enhances their optimistic expectations toward future learning and increases academic self-efficacy, ultimately elevating subjective wellbeing (Bandura, 1997; Blackwell et al., 2007; Yeager et al., 2019). Additionally, students with growth mindset proactively seek academic social support and build positive interpersonal relationships (Martin and Dowson, 2009), which further satisfies their need for relatedness. This fulfillment further promotes the enhancement of academic self-efficacy and intensifies experiences of wellbeing (Wentzel, 2012). In summary, a growth mindset fulfills the three basic psychological needs—autonomy, competence, and relatedness—which strengthen elementary school students' academic self-efficacy, and consequently elevate their subjective wellbeing.

### 4.3 The moderating role of perceived teacher support

This study found that perceived teacher support significantly moderates the first half of the mediation pathway—specifically, the relationship between growth mindset and elementary school students' academic self-efficacy, confirming H3. In particular, compared to low perceived teacher support, high perceived teacher support strengthens the positive predictive effect of growth mindset on academic self-efficacy. This finding aligns with the theory of growth mindset-context interactions (Yeager et al., 2019).

At its core, growth mindset involves the belief that abilities can be improved through effort and learning, but this belief requires positive external feedback and support to be reinforced (Yeager et al., 2019). When students perceive high levels of teacher support, they not only receive positive feedback and cognitive strategy guidance from teachers, which enhances their courage to face challenges and confidence to overcome difficulties (Zheng and Wang, 2017), but also draw strength from the resources and success experiences provided by teachers, thereby improving their ability to tackle challenges and achieve academic goals (Jones, 2020; Ma et al., 2023). Consequently, through verbal persuasion and vicarious experiences facilitated by teachers, students can continuously strengthen their academic self-efficacy (Bandura, 1997). Conversely, when elementary school students perceive low teacher support, the lack of a safety in the learning environment, coupled with the absence of positive external feedback and effective guidance strategies necessary for achieving goals, can trigger or exacerbate feelings of frustration and incompetence. This subsequently leads to a significant reduction in their academic self-efficacy (Wang and Degol, 2016; Yeager et al., 2019; Yu et al., 2022). Furthermore, even when students exert effort, the absence of adequate support may impede substantive progress. This lack of concrete success experiences makes it difficult for students to translate their belief in malleable ability into effective action, ultimately trapping them in the “helplessness trap” (Dweck and Yeager, 2019; Zhang et al., 2021). Thus, perceived teacher support essentially acts as a “contextual amplifier” for the relationship between growth mindset and academic self-efficacy in elementary school students. This means it exerts a catalytic effect in high-support contexts, while producing an inhibitory effect in low-support contexts.

### 4.4 Research implications and limitations

This study constructed a moderated mediation model to explore the relationship between growth mindset and elementary school students' subjective wellbeing, alongside the mediating role of academic self-efficacy and the moderating role of perceived teacher support. The findings offer theoretical guidance and empirical support for enhancing subjective wellbeing among elementary school students.

First, fostering growth mindset should be regarded as a critical component of elementary school education. Growth mindset can be cultivated, and a fixed mindset can be transformed into growth mindset (Blackwell et al., 2007; Cooley and Larson, 2018; Dweck, 2006). Previous research has shown that parents and teachers can effectively nurture growth mindset in students by conveying positive beliefs about failure, adopting process-oriented evaluation methods (Haimovitz and Dweck, 2016), focusing on students' learning processes (Sun, 2018), and implementing growth mindset interventions (Lin, 2022). Second, while fostering growth mindset, particularly among students with a fixed mindset, teachers should prioritize enhancing their academic self-efficacy. Strategies such as providing opportunities for success experiences, showcasing role models, offering timely positive feedback, guiding learning strategies, creating a supportive learning environment, conducting attributional retraining, and encouraging extracurricular physical activities (Bandura, 1997; McAuley et al., 2000; Olivier et al., 2019) can effectively boost students' academic confidence. Third, it is essential to emphasize the development of a teacher-supported environment. By cultivating a classroom culture that embraces growth mindset principles (Zhang et al., 2021), organizing growth-oriented class activities (Kroeper et al., 2022), implementing growth mindset teaching strategies (Boaler, 2015), and adopting positive instructional evaluation methods, educators can create fertile environment for cultivating of growth mindset.

However, this study has several limitations that warrant attention in future research. First, data relied exclusively on self-report questionnaires administered to elementary school students. Growth mindset, as a socially desirable construct, is susceptible to expectancy effects (Dweck, 2002). Moreover, compared to middle school and university students, elementary school students are more likely to hold an optimistic view of their perceived abilities, regardless of their actual performance (Muenks et al., 2018). Therefore, future research should collect data from multiple sources, such as parents, teachers, and peers, to mitigate the impact of social desirability bias on the validity and reliability of the findings. Second, although the theoretical model constructed in this study contributes to understanding the relationships among growth mindset, subjective wellbeing, and academic self-efficacy, it is fundamentally a cross-sectional correlational study and cannot establish causal relationships between variables. Future research could employ longitudinal designs or clinical intervention studies to further examine the causal relationships among these variables.

## 5 Conclusions

Growth mindset serves as a positive predictor of elementary school students' subjective wellbeing, with academic self-efficacy partially mediating this relationship. Notably, the association between growth mindset and elementary school students' academic self-efficacy is moderated by perceived teacher support, such that the positive effect of growth mindset on elementary school students' academic self-efficacy is more pronounced under high vs. low levels of perceived teacher support.

## Data Availability

The raw data supporting the conclusions of this article will be made available by the authors, without undue reservation.
